# Leloir Glycosyltransferases in Applied Biocatalysis: A Multidisciplinary Approach

**DOI:** 10.3390/ijms20215263

**Published:** 2019-10-23

**Authors:** Luuk Mestrom, Marta Przypis, Daria Kowalczykiewicz, André Pollender, Antje Kumpf, Stefan R. Marsden, Isabel Bento, Andrzej B. Jarzębski, Katarzyna Szymańska, Arkadiusz Chruściel, Dirk Tischler, Rob Schoevaart, Ulf Hanefeld, Peter-Leon Hagedoorn

**Affiliations:** 1 Department of Biotechnology, Delft University of Technology, Section Biocatalysis, Van der Maasweg 9, 2629 HZ Delft, The Netherlands; l.mestrom@tudelft.nl (L.M.); s.r.marsden@tudelft.nl (S.R.M.); u.hanefeld@tudelft.nl (U.H.); 2 Department of Organic Chemistry, Bioorganic Chemistry and Biotechnology, Silesian University of Technology, B. Krzywoustego 4, 44-100 Gliwice, Poland; marta.przypis@polsl.pl (M.P.); daria.kowalczykiewicz@polsl.pl (D.K.); 3 Biotechnology Center, Silesian University of Technology, B. Krzywoustego 8, 44-100 Gliwice, Poland; 4 Environmental Microbiology, Institute of Biosciences, TU Bergakademie Freiberg, Leipziger Str. 29, 09599 Freiberg, Germany; andre.pollender@ioez.tu-freiberg.de (A.P.); antje.kumpf@ruhr-uni-bochum.de (A.K.); dirk.tischler@rub.de (D.T.); 5 Microbial Biotechnology, Faculty of Biology & Biotechnology, Ruhr-Universität Bochum, Universitätsstr. 150, 44780 Bochum, Germany; 6 EMBL Hamburg, Notkestraβe 85, 22607 Hamburg, Germany; ibento@embl-hamburg.de; 7 Institute of Chemical Engineering, Polish Academy of Sciences, Bałtycka 5, 44-100 Gliwice, Poland; andrzej.jarzebski@polsl.pl; 8 Department of Chemical and Process Engineering, Silesian University of Technology, Ks. M. Strzody 7, 44-100 Gliwice Poland.; katarzyna.szymanska@polsl.pl; 9 MEXEO Wiesław Hreczuch, ul. Energetyków 9, 47-225 Kędzierzyn-Koźle, Poland; arkach@mexeo.pl; 10 ChiralVision, J.H. Oortweg 21, 2333 CH Leiden, The Netherlands; schoevaart@chiralvision.com

**Keywords:** glycosyltransferase, applied biocatalysis, enzyme cascades, chemoenzymatic synthesis, sugar chemistry, carbohydrate, Leloir, nucleotide

## Abstract

Enzymes are nature’s catalyst of choice for the highly selective and efficient coupling of carbohydrates. Enzymatic sugar coupling is a competitive technology for industrial glycosylation reactions, since chemical synthetic routes require extensive use of laborious protection group manipulations and often lack regio- and stereoselectivity. The application of Leloir glycosyltransferases has received considerable attention in recent years and offers excellent control over the reactivity and selectivity of glycosylation reactions with unprotected carbohydrates, paving the way for previously inaccessible synthetic routes. The development of nucleotide recycling cascades has allowed for the efficient production and reuse of nucleotide sugar donors in robust one-pot multi-enzyme glycosylation cascades. In this way, large glycans and glycoconjugates with complex stereochemistry can be constructed. With recent advances, LeLoir glycosyltransferases are close to being applied industrially in multi-enzyme, programmable cascade glycosylations.

## 1. Introduction

Enzymes were already used for the conversion of glycosides even before all stereochemical details of the known carbohydrates were assigned [1,2]. In 1837, a crude formulation of almonds containing hydroxynitrile lyases catalyzed the enzymatic hydrolysis of the glycoside amygdalin [3]. Moving almost two centuries forward, the largest volumetric biocatalytic industrial process is the application of glucose isomerase for the production of high fructose syrup for food and drink applications, producing fructose from glucose at 10^7^ tons per year [4]. The secret of the success of enzymes in the production or treatment of carbohydrates and glycosides is their exquisite stereo- and regioselectivity. The excellent selectivity of enzymes is required due to the diversity of structural features of carbohydrates [5], comprising d- and l-epimers, ring size, anomeric configuration, linkages, branching, and oxidation state(s). Since drug targets often exhibit specificity for all of these structural features, the production process should not contain any side-products to prevent undesired side-effects [6].

**Figure 1:**
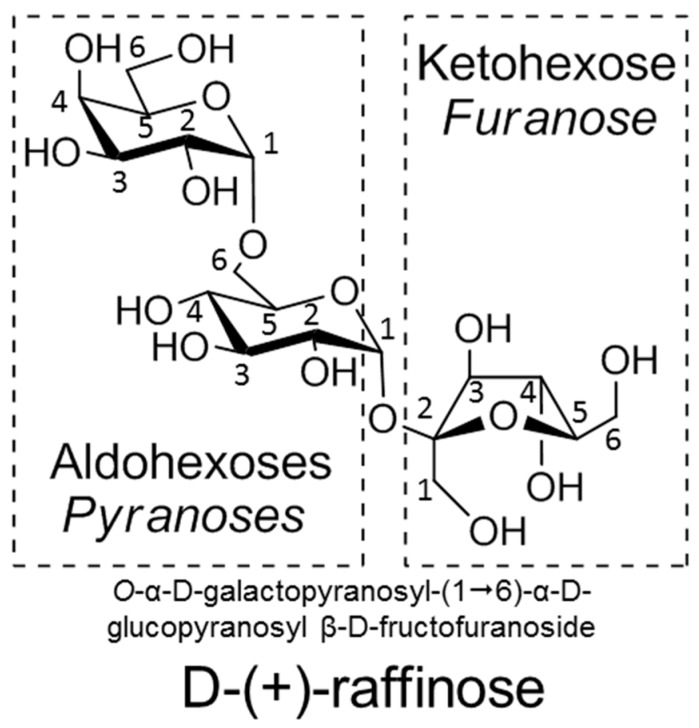
The nomenclature of glycosides and oligosaccharides.

The challenge in the synthesis of carbohydrates is their wide variety of functionalities and stereochemistry (Figure 1). (Poly)hydroxyaldehydes containing a terminal aldehyde are referred to as aldoses and (poly)hydroxyketones are defined as ketoses. In aqueous solutions, monosaccharides form equilibrium mixtures of linear open-chain and ring-closed 5- or 6-membered furanoses or pyranoses, respectively. For aldoses, the asymmetric ring forms at C-1. For ketoses, it closes at C-2 as an axial (α) or equatorial (β) hemiacetal or hemiketal, respectively (commonly defined as the anomeric center). A glycosidic linkage is a covalent *O*-, *S*-, *N*-, or *C*-bond connecting a monosaccharide to another residue resulting in a glycoside, while glucoside is specific for a glucose moiety. The equatorial or axial position of the glycosidic bond is referred to as α- (axial) or β-linkage (equatorial). The number of carbohydrates linked via glycosidic bonds can be subdivided into oligosaccharides with two to ten linked carbohydrates, while polysaccharides (glycans) contain more than ten glycosidic bonds. A glycan either contains multiple different monosaccharides or more than ten glycosidic bonds. A glycoconjugate contains at least one or more monosaccharides or oligosaccharides covalently attached to a non-carbohydrate moiety (aglycon). If an oligosaccharide contains an aldose or ketose that is in equilibrium with its open-chain form, the aldehyde or ketone can be oxidized with chemical reagents (e.g., with the Benedict reagent). This is referred to as the reducing end in oligosaccharides. If there is no possibility for the sugar to form the open chain-form, then this is called a non-reducing end. Non-reducing sugars are found in glycoconjugates (i.e. nucleotides) and oligosaccharides (i.e., raffinose).

**Scheme 1:**
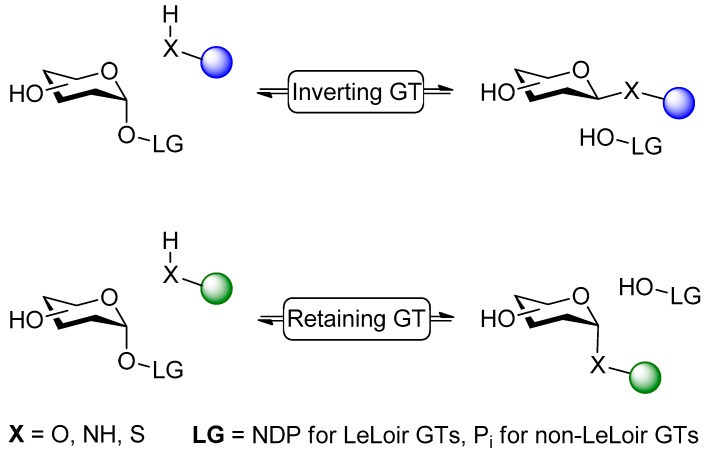
The overall scheme of an enzymatic glycosylation reaction for the biocatalytic synthesis of glycosides by retaining or inverting glycosyltransferases (GT) using NDP or P_i_ activated sugar donors for Leloir and non-Leloir GTs, respectively.

Glycosyltransferases (GTs) catalyze the transfer of a carbohydrate acceptor from an activated sugar nucleotide donor with high selectivity and yield, enabling the stereo- and regioselective extension and branching of large glycans and glycoconjugates (Scheme 1). Upon formation of the glycosidic bond, the stereochemistry can either be retained or inverted by GTs with high selectivity for the α- or β-anomer. Leloir glycosyltransferases utilize carbohydrates linked to a nucleotide diphosphate (NDP) with an α-linked glycosidic bond, where non-Leloir glycosyl transferase utilize a phosphorylated sugar donor. For both types of glycosyltransferase, the main driving force for the reaction to go to completion is the exergonic release of either P_i_ or NDP from their respective sugar donors. The choice of nucleotide acceptor determines the (stereo) chemical outcome of the type of *O*-, *NH*-, *S*-, *C*-glycosidic bonds.

The enzymatic treatment of glycosides is mainly applied in the food industry using non-LeLoir GTs, enhancing flavors and functionality in complex food formulations, such as debittering [7,8,9], sweetening [10,11], or clarification [12,13,14,15,16]. The high costs of nucleotides, enzymes, and (enzymatic) regeneration systems for the treatment or production of low-value carbohydrate-containing products limited the application of nucleotide-dependent LeLoir GTs within the industry in the past. However, recent advancements in glycobiology have sparked interest in the (chemo) enzymatic production of high-value glycosides and glycoconjugates with high yield and selectivity for pharmaceutical applications [17,18,19,20,21,22]. As more LeLoir GTs are being reported with high protein expression, wide substrate scope, and high selectivity, industrial enzymatic glycosylation for the production of glycosides and glycoconjugates in vitro is becoming economically feasible. For instance, the expression of a large part of the human glycosyltransferases is a new hallmark for the production of human glycans or glycoconjugates [23], simplifying their chemoenzymatic synthesis. Besides these developments, the reaction methodologies are currently being further optimized. Multi-step enzymatic coupling with glycosyltransferases using non-natural sugar acceptors and nucleotide sugar donors have been performed with automated synthesizers and are under development, as was recently reviewed [24].

The enzymatic synthesis of glycosides has received increasing attention in organic synthesis. However, the application of Leloir glycosyltransferases in a multi-enzymatic sugar coupling process is challenging from a process design point of view. The high costs, low stability, and difficult or limited availability of nucleotide sugar donors, in addition to the challenging protein production of Leloir glycosyltransferases hamper the development of enzymatic glycosylation. As a compromise, separate nucleotide sugar regeneration cascades and optimization of the protein production of industrial biocatalysts has been pursued [25]. Although there is a large body of scientific literature reporting on the biochemical properties and the reactions that glycosyl transferring enzymes catalyze, the performance of these biocatalytic processes has only sparingly been described. Due to their inherent complexity, kinetic and thermodynamic parameters have often not been analyzed in detail for the production of larger oligosaccharides using Leloir glycosyltransferases. In this review, the possibilities and limitations for industrial applications of Leloir glycosyltransferases are highlighted from the intersection of biochemical, chemical, thermodynamic, and reaction engineering perspectives, giving an overview of the requirements of industrial processes involving glycosyltransferases.

## 2. Glycosyltransferases in Nature

**Figure 2:**
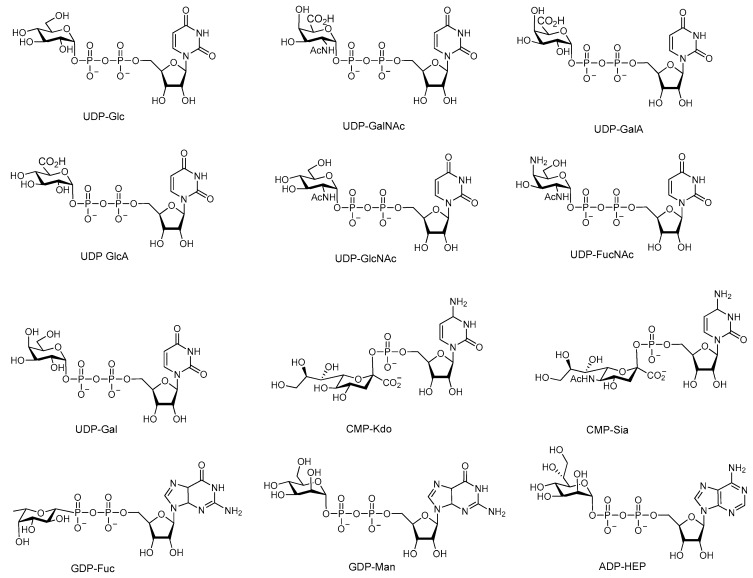
Common sugar nucleotides found in all kingdoms of life. Abbreviations: UDP-Glc, UDP-glucose; UDP-GalNAc, UDP-*N*-acetyl-2-deoxy-d-galactosamine; UDP-GalA, UDP-d-galacturonic acid; UDP-GlcA, UDP-d-glucuronic acid; UDP-GlcNAc, UDP-*N*-acetyl-2-deoxy-d-glucosamine; UDP-FucNAc, UDP-*N*-acetyl-l-fucosamine; UDP-Gal, UDP-d-galactose; CMP-Kdo, CMP-3-deoxy-d-manno-octulosonate; CMP-Sia, CMP-*N*-acetylneuraminic acid; GDP-Fuc, GDP-l-fucose; GDP-Man, GDP-d-mannose; ADP-HEP, ADP-l-*glycero*-d-*manno*-heptose.

Glycosyltransferases catalyze the formation of a glycosidic bond between an unactivated acceptor monosaccharide or aglycon and an activated sugar donor [26] to a di-, oligo-, polysaccharide [27], lipo(poly)saccharide [28] or peptidoglycan [29]. More than 484,620 glycosyltransferases in over 106 families have been identified according to the carbohydrate active enzymes (CAZy) database under the Enzyme Commission number E.C.2.4.x.y. (CAZy database, last updated 01/15/18 [30]), representing an enormous number of metabolic pathways [31]. Glycosyltransferases can be sub-classified based on four different criteria: (i) the class of substrates [32]; (ii) the protein structure [26]; (iii) the preference in stereochemistry [27,33]; (iv) the dependency on metals for catalytic activity [26]. Non-Leloir glycosyltransferases use phosphorylated donors (i.e., lipid polyprenol [34,35], sugar 1-phosphates [32]) and can be described as phosphorylases. The second class are transglycosidases accepting non-activated di- or polysaccharides as carbohydrate donors. The largest class of glycosyltransferases are the nucleotide-dependent Leloir glycosyltransferases [26,32,36], named in honor of Luis Federico Leloir, who received a Nobel prize for the discovery of nucleotide sugar donors in 1970 (Figure 2).

**Figure 3:**
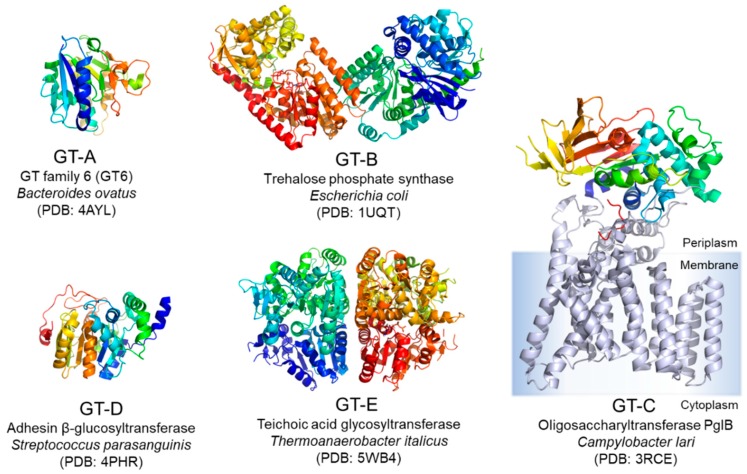
Protein folds of Leloir glycosyltransferases (GT-A, GT-B, GT-D) and non-Leloir glycosyltransferases (GT-C, GT-E).

The protein sequence and crystallographic data demonstrate that glycosyltransferases are mainly comprised of five different protein folds (Figure 3) [26,27,37]. Glycosyltransferases having a GT-A or GT-B fold consist of two β/α/β-Rossmann-like domains, abutting each other in case of the GT-A fold or facing each other for GT-B folds [26,27,38]. Both folds contain separate donor and acceptor binding sites [26]. Gloster et al. reported that glycosyltransferases with a GT-A fold belong to the divalent metal ion dependent class of these enzymes, whereas GT-B folds are often metal ion independent [26,27]. Interestingly, glycosyltransferases having a GT-C fold are non-Leloir glycosyltransferases, utilizing membrane integrated or membrane linked proteins with lipid phosphate sugar donors, also known as non-Leloir donors [27,32,37]. The Leloir glycosyltransferases containing a GT-D fold catalyze the transfer of glucose to hexasaccharide *O*-linked to serine-rich repeats of bacterial adhesins [39]. The most recent addition, is the *N*-acetyl-d-mannose transferase utilizing non-Leloir undecaprenyl-linked glycosyl diphosphates with a unique GT-E fold [40].

**Figure 4:**
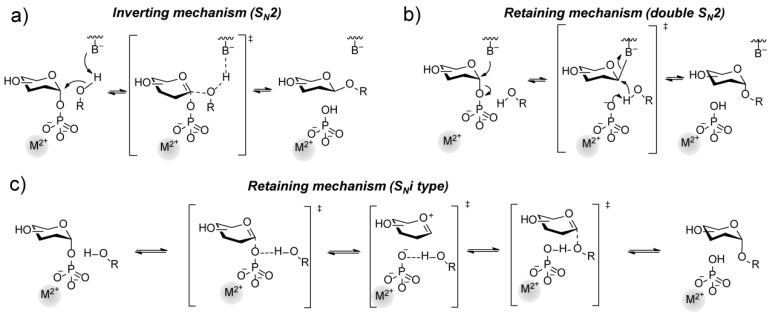
Reaction mechanism of glycosyltransferases upon inversion (**a**) or retention (**b**,**c**) of the anomeric glycosidic bond. The divalent metal (M^2+^) is not necessarily a requirement for catalytic activity for GTs.

For Leloir glycosyltransferases, the binding of the sugar donor nucleotide and acceptor follows a sequential ordered bi-bi catalytic mechanism via non-covalent interactions of the sugar donor nucleotide. The binding of the sugar or aglycone acceptor results in an enzyme-substrate ternary complex [41]. Hydrolysis of the sugar nucleotide donor is prevented by the tight binding in an unproductive state, where the high affinity of the enzyme for the sugar nucleotide donor is an indicator for product inhibition (K_i_) by the released nucleotide [42]. For Leloir glycosyltransferases, a lower affinity or promiscuity towards the nucleotide donor results often in less product inhibition [43]. Upon binding of the sugar nucleotide donor, the enzyme undergoes a conformational change stabilizing the transition state, resulting in the formation of a glycosidic bond and the release of the nucleotide donor. Different reaction mechanisms of glycosyltransferases have been described and reviewed [26,27,44,45,46,47]. The *inverting* occurs via a S_N_2 mechanism, while a *retaining* transfer can proceed via a concerted or ion-pair intermediate mechanism through a double displacement via a S_N_2 mechanism. Also, a *transient covalent* intermediate via a S_N_i-type mechanism has been described for LeLoir GTs (Figure 4). *Inverting* glycosyltransferases use general base catalysis (i.e., aspartate or glutamate) [31,48,49] to form an oxocarbenium ion-like transition state. They show a catalytic rate enhancement by utilizing divalent metals (i.e. Mn (II) or Mg (II)), which are often coordinated by the amino acid motif Asp-X-Asp.

### 2.1. Distinguishing Glycosyl Transferases from Glycoside Hydrolases

**Figure 5:**
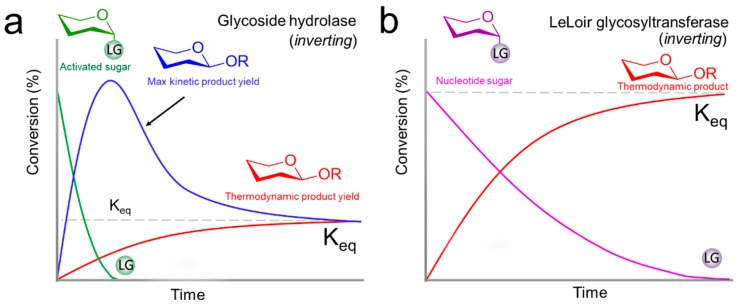
Exemplary enzymatic glycosylation of an activated sugar donor (green) and acceptor (R-group) to afford a maximum transient kinetic (blue) product yield catalyzed by a glycoside hydrolase, followed by reverse hydrolysis towards the thermodynamic product concentration. Direct esterification leads to the thermodynamic product yield K_eq_ without the requirement for an activated sugar (red) in (**a**). LeLoir GTs only catalyze the direct esterification of a nucleotide sugar donor (purple) to thermodynamic product (red) in (**b**).

Two main groups of enzymes can catalyze the regio-, stereo-, and enantioselective coupling of carbohydrates. *Glycoside hydrolases* and *glycosyltransferases* are often combined in biocatalytic retro-synthetic strategies for linear elongation and branching of oligosaccharides. Glycoside hydrolases are enzymes that condense a sugar donor with an aglycone acceptor. The broad substrate scope of glycoside hydrolases has resulted in numerous synthetic applications such as synthesis [50,51,52,53] or hydrolysis [54,55,56,57] of glycosidic bonds, and desymmetrization [58]. As a drawback, their broad substrate scope also leads to the formation of side-products. Glycosylations with glycoside hydrolases are under kinetic (transglycosylation) or thermodynamic control (direct glycosylation) using activated and non-activated sugars respectively (Figure 5). With *transglycosylation*, relatively high yields can be obtained in comparison with *direct glycosylation* due to a thermodynamically unfavorable reaction equilibrium (K_eq_) in water. As a rule of thumb, transglycosylation should be faster than glycoside hydrolysis, as otherwise the activated sugars would hydrolyze before the glycosylation reaction is completed. Also, the rate of hydrolysis of the product should be slower than the activated glycosyl donor or the product yield decreases. As this is often not the case, an excess of the activated sugar donor is required under kinetic control. Similar to the coupling of protected glycosyl donors, the donors for transglycosylation, such as fluoro [59,60,61,62], -azido [63], *p*-nitrophenyl- [64] or *p*-nitropyridyl- [65], vinyl- [66], and allyl-glycosides [67] require their separate synthesis. The direct glycosylation is challenging due to the poor K_eq_ under aqueous reaction conditions, limiting the degree of conversion. The product yields with direct glycosylation can be improved by adding one substrate in excess, lowering the water activity [68], and in situ product removal [69].

Leloir glycosyltransferases couple NDP sugar donors with a wide range of sugar acceptors resulting in the formation of a glycosidic bond. The exclusion of hydrolysis activity of the nucleotide sugar donor separates glycoside hydrolases from glycosyltransferases. Nevertheless, hydrolysis of the nucleotide sugar donor in the absence of a sugar acceptor has been reported and is referred to as “error hydrolysis” [70,71,72,73]. Hence, the competition between water or a sugar acceptor as nucleophile is important for the efficiency of glycosylation. Only a handful of studies investigated the nature of the hydrolysis activity of Leloir glycosyltransferases with sugar nucleotide donors. For instance, the bacterial sialyltransferase from *Pasteurella dagmatis* hydrolyzed the rather hydrolysis-prone CMP-Neu5Ac in the absence of another substrate [74]. Directed evolution has been shown to be an effective tool to diminish the degree of hydrolysis of NDP sialyl donor [75,76]. In comparison to hemiketals, hemiacetals are more stable sugar donor nucleotides (i.e., GDP-l-fucose). Here, the Leloir glycosyltransferases catalyze hydrolysis to a lesser degree [77]. Interestingly, the affinity of water to the active site for the hydrolysis of sugar nucleotide donors has not been determined for Leloir glycosyltransferases.

### 2.2. Recombinant Expression of Glycosyl Transferases

Although protein structures and the reaction mechanism of Leloir glycosyltransferases are widely investigated, production of the enzyme is often challenging. Heterologous bacterial hosts such as *E. coli* often lead to poor expression or formation of inclusion bodies (IBs) in certain cases with retention of catalytic activity [78,79]. Besides the difficulties in recombinant protein production and isolation, the half-life of this class of enzymes is often less than a couple of hours [80,81,82]. Thermostable glycosyltransferases from thermophilic archaea show higher overall stability [43]. Leloir glycosyltransferases are often aggregation-prone in vitro [83,84]. As a solution to their aggregation, a large number of solubility tags have been successfully applied to increase the solubility of Leloir glycosyltransferases [23,79,85,86]. The recent advance of using the fluorescent proteins mCherry [79] or GFP [23] as tags allowed for both an increase in solubility as well as rapid protein quantification. For example, the fusion of GFP allowed for a modular expression approach of all human glycoenzymes in HEK293 cells enabling multi-milligram isolation from the culture media in 65% of all cases [23].

The optimization of protein expression, the number of enzymes discovered, and the characterization of a wide range of Leloir GTs has led to fundamental insights into their protein structures, reaction mechanism, and substrate spectrum. The result of this extensive biochemical knowledge is leading to the adoption of Leloir glycosyltransferases within the field of carbohydrate chemistry. Next, we will discuss how these biochemical insights have been developing alongside their application in chemoenzymatic glycosylations of glycoconjugates and oligosaccharides.

## 3. Application of Glycosyl Transferases in Organic Synthesis

The production of glycosides and glycans requires the use of highly selective catalysts to prevent the formation of side-products. The development of automated chemical methods such as the solid-phase production of oligosaccharides using the Seeberger method [87], the Demchenko synthesizer using HPLC-based platforms for automation [88], and the Yoshida procedure employing an electrochemical oxidation step [89], improved glycochemistry significantly. The basic principle of elongating a sugar on a solid particle by performing a coupling-wash-deprotection-wash cycle under computer control allows for the rapid production of a wide variety of carbohydrates [90,91,92,93,94,95,96,97]. The mechanism of action is the assembly of an oligosaccharide using protection group manipulation of either an activated glycosyl acceptor or donor [98,99,100,101,102,103,104,105]. The purification of the intermediates produced in sequential reactions remains the largest hurdle for chemical synthesis of an oligosaccharide or glycan. In particular, the low orthogonality of activated glycosyl donors and acceptors limits multiple glycosylation reactions in one-pot reactions. Also, the inherently low chemical reactivity of certain glycosidic bond forming reactions, such as α-sialylation [106,107,108] and β-mannosylation [109], restrict different types of linkages. Enzymes which catalyze one-pot glycosylation reactions with unprotected sugars can produce different types of glycosidic linkages and have expanded the synthetic toolbox of glycochemistry considerably.

Leloir glycosyltransferases (GTs) transfer a nucleotide sugar donor to an aglycon acceptor, forming *O*-, *N-* [110,111,112,113,114,115,116,117,118,119,120,121,122,123,124] or the rare *C*- [125,126,127,128,129,130,131,132,133,134,135,136] and *S*-glycosidic bonds [137,138,139,140] under thermodynamic control. In comparison to chemical methods, the enzymatic coupling of carbohydrates occurs without the use of protecting groups in a highly selective manner, allowing for orthogonal one-pot multi-enzymatic (OPME) reactions. With a few robust GTs, complete libraries of glycans can be constructed [141,142,143], which is particularly interesting since most of the human GTs are accessible in heterologous expression systems [23]. The advantages of employing GTs are their mild reaction conditions, short reaction times, pH tolerance, high specific activity, and high yields allowing for the (poly)glycosylation of a wide array of glycans.

### 3.1. Catalytic Reversibility of Glycosyltransferases

One of the notable discoveries on glycosyltransferases was the recognition that glycosyltransferases do not catalyze unidirectional reactions [144]. Alternatively, synthetic sugar donors and/or (chemo) enzymatic regeneration systems either alter the overall K_eq_ or regenerate the nucleotide in situ [145,146,147,148,149]. Such regeneration systems are not always a requirement; the glycosylation with nucleotide sugar donors allows for repeated glycosylation on a single aglycon (i.e., flavonol-*O*-diglycoside [150]) or elongation of a (poly)saccharide, such as glycogen with a molecular weight of up to 10^7^ kDa [151]. The high glycosylation efficiency with Leloir GTs arises from a favorable thermodynamic equilibrium K_eq_ in these examples, determined by the sugar nucleotide donor and carbohydrate or aglycone acceptor, pH, and ionic strength. As mentioned earlier, in a few examples the hydrolysis of the NDP-sugar donor has been reported for Leloir GTs [70,71,72,73,74,75,76,77]. In these particular cases, it is important to emphasize that the glycosylation with Leloir GTs is under kinetic control, and the sugar acceptor and water are competing nucleophiles throughout the entire course of reaction [70,71,72,73,74,75,76,77].

**Figure 6:**
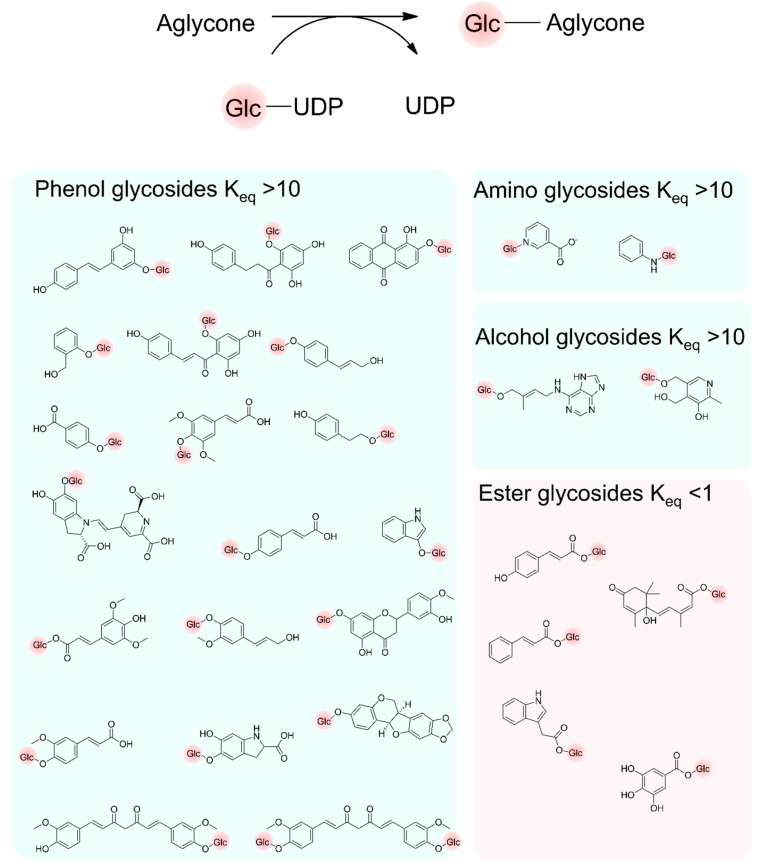
Glycosylation of aglycones producing phenolic glycosides, amino glycosides, alcohol glycosides, ester glycosides, and disaccharides with their estimated K_eq_. The K_eq_ was calculated from the Gibbs free energy ΔG_r_′° using the eQuilibrator web interface (http://equilibrator.weizmann.ac.il) [155] assuming the following conditions: ionic strength 0.1 M, pH 7.0, aglycon (1 mM), UDP (1 mM), UDP-d-glucose (1 mM), glycosylated product (1 mM), and 298 K.

A large impact on the field of glycobiology is the improved group estimation method [152,153] for the determination of the change in Gibbs free energy of formation of glycosylation reactions with increased accuracy, named eQuilibrator 2.0 [154,155]. In comparison to empiric thermodynamic data (i.e., Thermodynamics of Enzyme-Catalyzed reactions Database [156]), prediction tools allow for a much higher coverage of Gibbs free energies of formation for different compounds. As a drawback, such prediction methods can lead to contradictory observations due to either experimental uncertainties [157] or incorrect analysis of given data [158]. Using Equilibrator 2.0, the synthesis of naturally occurring glycosides with nucleotide diphosphates (NDPs) were shown to be thermodynamically favorable, as is known for the glycosylation of phenolic [159,160,161,162,163,164,165,166,167,168,169,170], amino [171,172], or alcoholic [173] aglycones (Figure 6), and has been reviewed recently [44]. Interestingly, the importance of the pH has been reported for the glycosylation of acids [167,174,175] resulting in a low K_eq_ < 1 at a neutral pH. The K_eq_ depends on the pK_a_ of the aglycone- or saccharide acceptor, as well as the terminal phosphate of the sugar nucleotide donor.

### 3.2. Sugar Donors and Acceptors and Their Glycosylation Efficiency

The thermodynamic constraints of enzymatic glycosylations of sugar acceptors with nucleotide donors for the synthesis of di-, oligo-, or polysaccharides has been explored to a lesser extent. Sucrose synthase has been employed for the regeneration of nucleotide sugars [25,42,80,176,177]. The equilibrium constant (K_eq_) of the reaction of sucrose with UDP to afford the sugar donor UDP-glucose was determined [178]. The pH influences the K_eq_ for the synthesis of UDP-glucose with *Acidithiobacillus caldus* sucrose synthase (*Ac*SuSy) due to the (de)protonation of the phosphate group of the NDP: going from a pH of 5.0 to 7.0 lowered the K_eq_ of 1.14 to less than 0.1 [178]. Enzymatic regeneration of NDP-glucose can also be achieved using trehalose as substrate [179]. For the regeneration of nucleotide sugars, sucrose has been described as a more attractive d-glucopyranosyl donor than α, α-d-trehalose due to the lower free energy of the glycosidic bond, resulting in a more favorable thermodynamic equilibrium [44,180].

While the type of carbohydrate donor and acceptor determines the glycosylation product, the respective choice of nucleotide used for activation of the donor glycoside is important from a thermodynamic point of view. Similar enzyme activities and affinities were observed for the coupling of UDP-, GDP-, and ADP-glucose with α-d-glucose by trehalose transferase (TreT) from *Pyrococcous horikoshii*. However, different K_eq_ were observed for the enzymatic production of d-trehalose. [43] In line with these observations, a trehalose transferase from *Thermoproteus uzoniensis* fused to a mCherry solubility tag also reported different K_eq_ for ADP- and UDP-glucose for the production of d-trehalose [79]. Hence, the overall extent of conversion for the synthesis of disaccharides was determined by the thermodynamics of the nucleotide. Although a thorough examination of the Gibbs free energy of formation of NMP, NDP, or NTP salt or metals pairs in aqueous solution is beyond the scope of this review, it should be noted that the pKa of nucleotides differ affecting the Gibbs free energy of formation. Indeed, the ADP/ADP-glucose couple shows the largest Gibbs free energy change for a transfer of α-glucopyranosyl moiety to a nucleotide, followed by UDP, CDP, and dTDP according to Equilibrator 2.0 [155]. Nature might evolve enzymes to catalyze either the synthesis of nucleotide sugar donors or reactions based on the K_eq_ of nucleotides, as TreT of *Thermococcus litoralis* solely accepts ADP for the transfer of an α-glucopyranosyl moiety from trehalose to produce ADP-glucose [181]. Oppositely, TreT from *Thermoproteus tenax* utilizes UDP-glucose for the synthesis of trehalose since UDP favors synthesis [182]. Further work regarding this is required to elucidate the nature of the effect of nucleotides on the K_eq_ in a more comprehensive manner.

**Figure 7:**
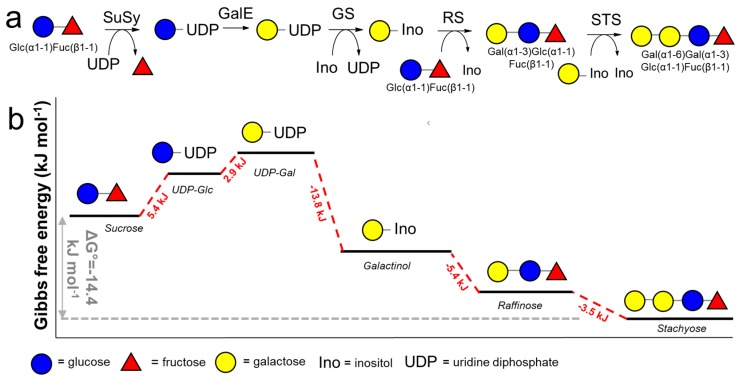
Enzymatic cascade for the production of stachyose from sucrose with glycosyltransferases. (**a**). The standard Gibbs free energy changes of the individual reactions (ΔG°s, red) and the total reaction (ΔG°, grey) shown in (**b**) [183]. The Δ_r_G′° represents the change of Gibbs free energy and was calculated using the eQuilibrator web interface (http://equilibrator.weizmann.ac.il) [155] using the following conditions: ionic strength 0.1 M, pH 7.0, 1 mM of component, 298 K. Abbreviations: UDP-d-glc, UDP-d-glucose; UDP-d-gal, UDP-d-galactose, SuSy, Sucrose synthase; GalE, UDP-d-glucose-4-epimerase; GS, galactinol synthase; RS, raffinose synthase; STS, stachyose synthase.

Under thermodynamic control, Leloir glycosyltransferases produce oligosaccharides if the overall glycosylation reaction is exergonic (Figure 7). A one-pot procedure using five enzymes allowed for the production of raffinose and stachyose from sucrose [183], using unpurified cell-free extract formulations and supplementation of UDP with a total-turnover number (TTN) of 337. Thermodynamic constraints were observed in the endergonic nucleotide sugar donor production, while coupling of the galactinol, raffinose, and stachyose were exergonic, thereby driving the overall reaction toward oligosaccharide synthesis. The estimation of the Gibbs free energy of individual components gives insights into energetic constraints of one-pot multi-enzyme Leloir glycosyltransferase catalyzed glycosylation reactions. An understanding of these limitations is essential for the optimization of industrial process conditions and reactor design (i.e., product removal) of a biocatalytic process.

### 3.3. NTP Regeneration for NDP-Sugar Donor Production

Recycling of the nucleotide sugar donor is considered essential for the application of Leloir glycosyltransferases in large scale applications by preventing product inhibition from the released nucleotide and reducing costs of expensive nucleotides. The use of purified enzymes in comparison to whole-cell systems is often preferred, due to undesired side-reactions of endogenous enzymes of the recombinant hosts during glycosylation of complex oligosaccharides. Most of the glycosyltransferases and enzymes involved in the regeneration of nucleotides operate under neutral conditions and often require the presence of divalent metals, such as Mg^2+^ or Mn^2+^. As Leloir glycosyltransferases use the elimination of the nucleotide as a driving force for the glycosylation reaction, the high energy gain poses a problem during the regeneration of NDP-sugar donors. For the production of NTP, the driving force then has to be derived from even more energy-rich donors.

**Figure 8:**
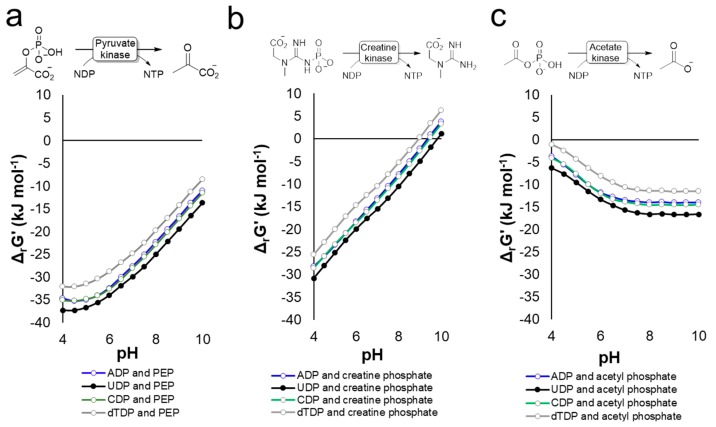
The use of different energy-rich phosphate donors to regenerate NTP using either pyruvate- (**a**), creatine- (**b**), or acetate kinase (**c**). The Δ_r_G′° represents the standard change of Gibbs free energy and was calculated using the eQuilibrator web interface (http://equilibrator.weizmann.ac.il) [155] using the following conditions: ionic strength 0.1 M, pH 7.0, 1 mM of component, 298 K. Abbreviations: NDP, nucleotide diphosphate; NTP, nucleotide triphosphate; ADP, adenosine diphosphate; UDP, uridine diphosphate; CDP, cytidine diphosphate; dTDP; deoxythymidine diphosphate; PEP, phosphoenolpyruvate.

Four of the most widely applied enzymatic methods for the regeneration of the nucleotide triphosphates (NTPs) are (see Figure 8): (1) pyruvate kinase using phospho(enol)pyruvate (PEP), (2) acetate kinase using acetyl phosphate, (3) creatine kinase using creatine phosphate, and (4) polyphosphate kinase using polyphosphate. The reaction equilibrium for PEP is highly favorable and the phosphate donor is stable in solution [184]. However, commercial phosphoenolpyruvate is expensive. Creatine phosphate is an alternative donor which is more affordable, but has considerably lower energetic advantages than PEP. A cheap energy-rich phosphate donor is acetyl phosphate, which can be synthesized directly from acetic anhydride and phosphate in excellent yields [185,186]. The disadvantage of using acetyl phosphate is the rapid spontaneous hydrolysis in water, requiring either continuous supplementation or an excess of acetyl phosphate. The inexpensive (poly)phosphate is a linear polymer that contains from ten to hundreds of energy-rich phosphate linkages [187]. (Poly)phosphate can drive the glycosylation reaction towards completion by the exergonic cleavage of the phosphoanhydride bond (ΔG° = −30 − −32 kJ·mol^−1^ [158]) upon phosphorylation of nucleosides with polyphosphate kinase (PPK). Mono- or diphosphorylation with PPK have been reported for ATP [184,188,189,190,191,192,193], UTP [189,194,195], CTP [196,197], tTMP [198], often showing broad promiscuity towards different nucleotides [187].

**Figure 9:**
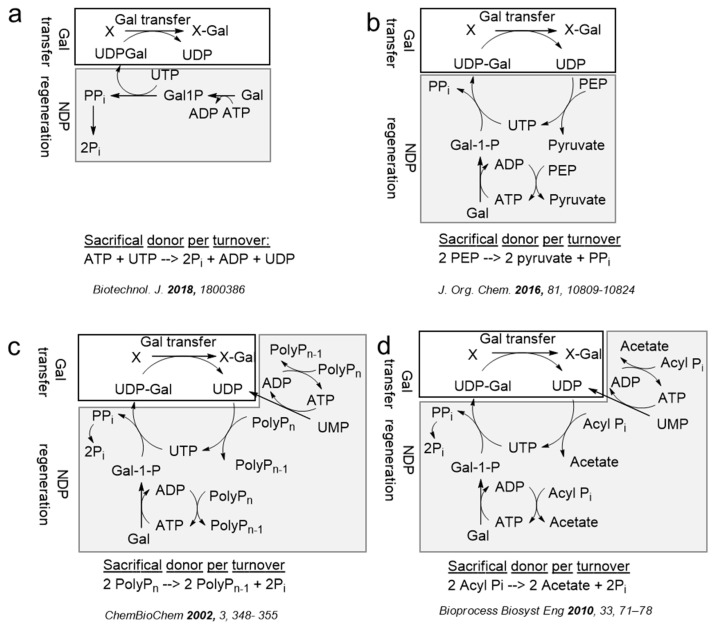
Several sacrificial phosphate donors for sugar nucleotide (re)generation systems of galactosyltransferases using a stoichiometric amount of NTPs (**a**) [199], PEP (**b**) [200], PolyP_n_ (**c**) [189], and acyl P_i_ (**d**) [201]. Abbreviations: PP_i_, pyrophosphate; P_i_, orthophosphate; UMP, uridine monophosphate; UDP, uridine diphosphate; UTP, uridine triphosphate; UDP-Gal, UDP-d-galactose; ADP, adenosine diphosphate; ATP, adenosine triphosphate; Gal, d-galactose; Gal1P, d-galactose-1-phosphate; PEP, (phospho)enol pyruvate; PolyP_n_, (poly)phosphate; acyl P_i_, acetyl phosphate.

Different (re)generation schemes for the in-situ production of nucleotide sugars for the transfer of a galactosylpyranoside moiety with either stoichiometric amounts of NTP [199], PEP [200], poly(phosphate) [189], or acetyl phosphate [201] are shown in Figure 9. The main driving force for the glycosylation reaction is the exergonic hydrolysis of pyrophosphate to phosphate by pyrophosphatases or alkaline phosphatases. Although it has been suggested that the sacrificial hydrolysis of NTPs with alkaline phosphatases is beneficial due to the removal of the nucleotide mono-, di-, or triphosphate inhibitors [44,202], experimental evidence separating thermodynamics (additional hydrolysis of pyrophosphate) from kinetics (product inhibition) is often not investigated in detail. It is evident that under thermodynamic control nucleotide regeneration and enzymatic glycosylation can only occur with highly exergonic sacrificial substrates (i.e., hydrolysis of pyrophosphate), as was proposed by Hirschbein et al. who compared the energy of hydrolysis of the sacrificial donors as a rationale for glycosylation efficiency [203]. Besides, for the common nucleotide glycosylation donors UDP-Glc, UDP-GlcNAc [204], UDP-GlcA [205], UDP-Gal [204], UDP-GalA, UDP-Xyl, GDP-Man, GDP-Fuc [204], CMP-Neu5Ac [200,204] the (re)generation systems for the production have been employed for rare or synthetic nucleotide sugar donors, such as CMP-MAnNGc [200], CMP-Man [200], CMP-ManNac5OMe [200], CMP-Kdo [200], ADP-Hep [206], and dTDP-Rha [207].

**Figure 10:**
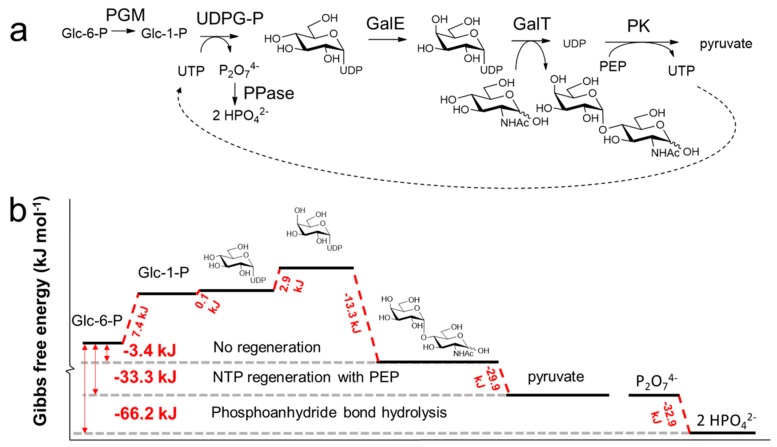
Enzymatic glycosylation for the production of *N*-acetyl-d-lactosamine from glucose-6-phosphate and *N*-acetyl-d-glucosamine (**a**) [208]. The Δ_r_G′° represents the standard change in Gibbs free energy (**b**) was calculated using the eQuilibrator web interface (http://equilibrator.weizmann.ac.il) [155] using the following conditions: ionic strength 0.1 M, pH 7.0, 1 mM of component, 298 K. Abbreviations: Glc-6-P, d-glucose-6-phosphate; Glc-1-P, d-glucose-1-phosphate; P_2_O_7_^4−^, pyrophosphate; HPO_4_^2−^, orthophosphate; UDP, uridine diphosphate; PGM, Phosphoglucomutase; UDPG-P, UDP-glucose pyrophosphorylase; GalE, UDP-galactose epimerase; GalT, galactosyltransferase; PK, pyruvate kinase.

Often, one-pot multienzyme (OPME) cascade reactions do not go to completion without the thermodynamic driving force from in-situ regeneration systems of nucleotide sugar donors. For instance, the gram-scale OPME cascade of the glycoconjugate *N*-acetyl-d-lactosamine resulted in 85% isolated yields and TTN of 80 for UTP (Figure 10a) [208]. The PEP/UDP-regeneration system produces UTP at the expense of PEP while the hydrolysis of pyrophosphate provides the thermodynamic driving force to complete the glycosylation cycle (Figure 10b). Upon replacement of the PEP/UDP-regeneration system with (poly)phosphate/UDP for the enzymatic production of *N*-acetyl-d-lactosamine, no additional pyrophosphatases are required [195]. Here, the required driving force is generated by the hydrolysis of the energy rich phosphoanhydride bond in (poly) phosphate instead of pyrophosphate hydrolysis.

Besides enzymatic regeneration, the enzymatic NTP synthesis can be performed directly from nucleosides in the presence of an excess of a phosphate donor reducing the overall costs of reagents (i.e., less than US$10 per gram UTP). A mutant of uridine kinase from *Thermus thermophilus* phosphorylates a broad range of nucleosides [209]. The addition of an excess of acetyl phosphate allows for the phosphorylation of nucleosides to NMPs with lysates from recombinant *E. coli* containing the overexpressed and promiscuous uridine kinase [207]. Advantageously, cell-free extracts from *E. coli* contain naturally occurring kinases that catalyze sequential phosphorylations to NTP in high yields. Recently, a recombinant *E. coli* strain containing an enzymatic cascade of eight enzymatic steps showed promising production titers of 1.4 g UTP per liter within 2.5 h starting from uracil [210]. Such regeneration systems have been extended to non-natural nucleosides [207,211,212].

### 3.4. Chemoenzymatic NTP Regeneration Cascades

The chemoenzymatic synthesis of NDP-sugar donors has been investigated with activated glucose donors under kinetic control. Although the realization that glycosyltransferases catalyze the reverse reaction dates back to 1957 [213], the first application for the glycosylation of nucleotides with an activated glycosyl fluoride was reported much later in 1999 [214]. The use of β-glucosyl fluoride for the production of UDP-α-glucose using a flavonoid *O*- and *C*-β-glycosyltransferases has been successful for the production of 3′-β-*C*-glucosylated phloretin under kinetic control [215]. Disadvantageously, fast hydrolysis of β-glucosyl fluoride in water limits its practical application. The pioneering work using nitrophenol glycosides demonstrated the broad adaptability of activated sugar acceptors for the glycosylation of nucleotide donors by altering the thermodynamics of the reaction [216]. The engineered inverting macrolide-inactivating glycosyltransferase (OleD) from *Streptomyces antibioticus* accepts a wide range of conveniently synthesized aromatic β-d-glucopyranoside donors for the production of UDP-α-d-glucose in the presence of UDP [145]. The directionality of the reaction is dependent on the nitrophenol β-d-glucopyranoside donor, ranging from exergonic favoring UDP-sugar formation to endergonic favoring the production of the aromatic sugar donor [145]. Alternatively, by coupling the 2-chloro-4-nitrophenol glycosides to catalytic amounts of nucleotide diphosphate, the glycosylation of a wide variety of substrates has been demonstrated [217,218,219]. However, the undesired hydrolysis of 2-chloro-4-nitrophenol glycosides by Leloir glycosyltransferases was observed as well [149]. Hence, separating glycosyltransferase from glycoside hydrolase activity is not always evident in Leloir glycosyltransferases.

### 3.5. One-Pot Multi Enzyme Cascades

**Figure 11:**
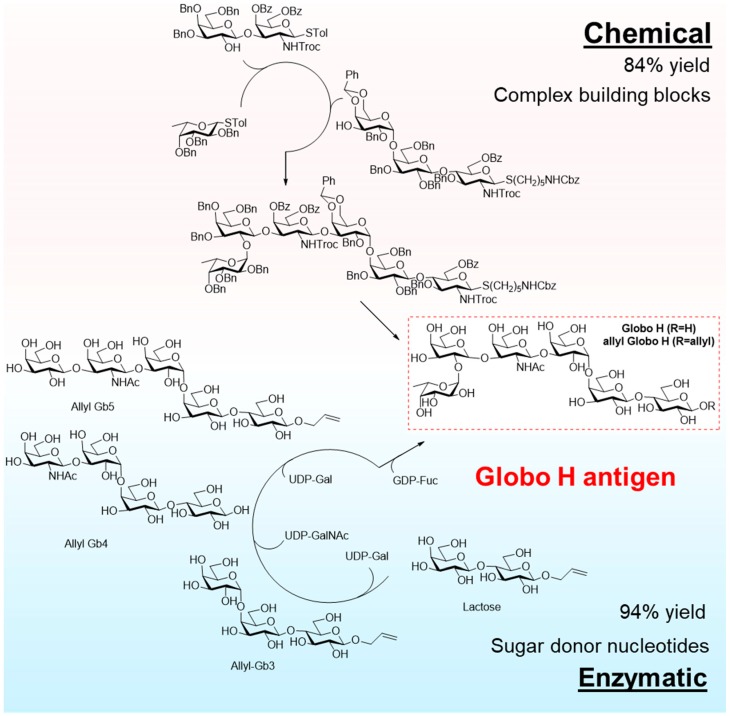
Reaction scheme of both the multistep chemical glycosylation utilizing previously synthesized carbohydrate building blocks [221] and enzymatic glycosylation utilizing nucleotide sugar donors for linear saccharide elongation capped with a terminal vinyl group [129]. The top half of the figure was adapted from [221], copyright 2006, National Academy of Sciences.

The use of a wide variety of NDP sugar donor regeneration systems coupled to exergonic sacrificial P_i_ donors inspired the extension of OPME systems towards oligosaccharides and glycans. Key to the success of Leloir glycosyltransferases is the selection of glycosyltransferases with high selectivities towards their substrates, and avoiding the formation of side-products. The human cancer antigen Globo H is a neutral hexasaccharide glycosphingolipid, which has been synthesized chemically by a linear sequence of 11 synthesis steps with predesigned building blocks resulting in a 2.6% overall yield [171]. The optimization of chemical glycosylation using the OptiMer program with custom-synthesized carbohydrate building blocks constructed Globo H in three consecutive steps, with an isolated yield of 41% [220]. The OptiMer program was improved to 83% isolated yield by a one-pot approach using a complex carbohydrate building block containing a Galα1-4Gal bond from a multi-step synthetic route [221], as is shown in Figure 11. A one-pot biocatalytic coupling of readily available nucleotide sugar donors UDP-galactose, UDP-*N*-acetyl-d-glucosamine, and GDP-fucose with three glycosyltransferases resulted in 54% isolated yield without any nucleotide sugar donor regeneration cycles [222]. Additional regeneration of the nucleotide sugar donor improved the overall yield to 94% at large-scale (i.e. 4.5 g allyl Globo-H) [129]. The efficiency of enzymatic glycosylation, the availability of the nucleotide sugar donors, the simplicity of the one-pot reaction, and the mild reaction conditions demonstrate the effectiveness of Leloir glycosyltransferases as catalysts for the production of complex saccharides.

**Figure 12:**
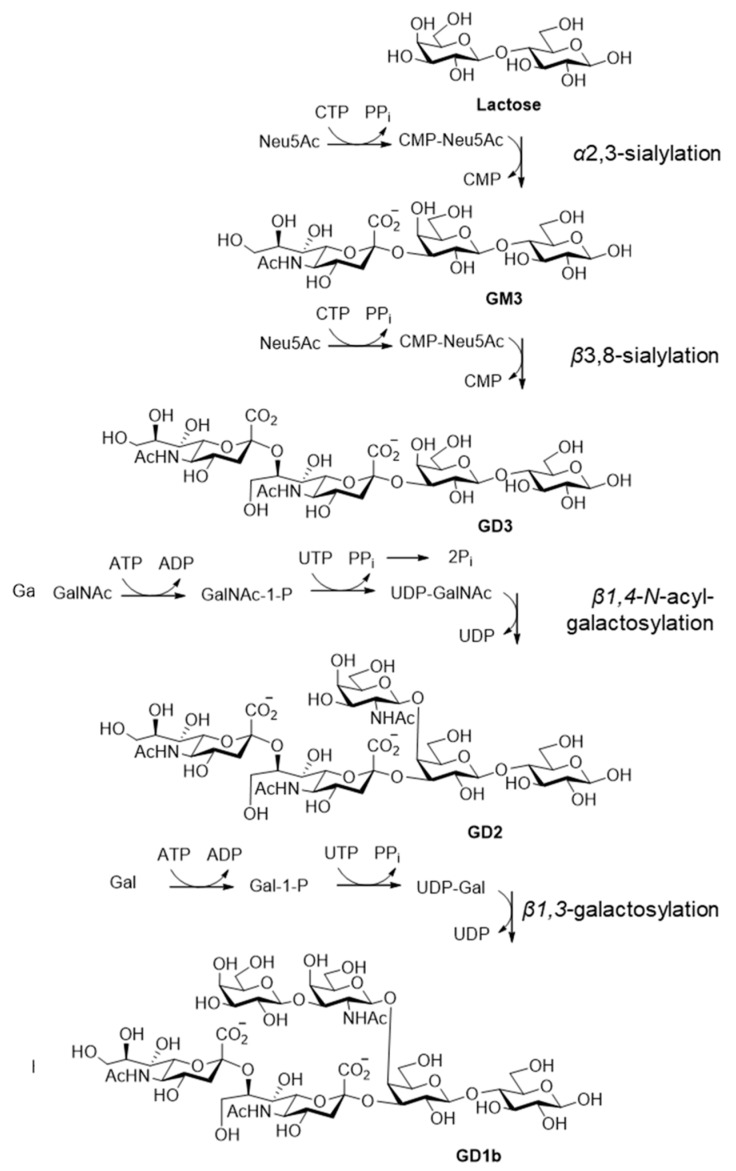
The enzymatic synthesis of GD1b glycan using OPME α2,3-sialylation, α3,8-sialylation, β1,4-*N*-acyl-galactosylation, and β1,3-galactosylation with a sacrificial (re)generation system for *N*-acetylneuraminic acid (Neu5Ac), *N*-acetylgalactosamine (GalNAc), and d-galactose (Gal).

Sequential OPME synthesis allows for the coupling of glycosidic linkages which are synthetically challenging, such as sialylation. The production of disialoganglioside cancer antigens GD1b and its derivatives by two sequential α-sialylation reactions has been performed using an engineered Leloir glycosyltransferase [200] with limited nucleotide donor hydrolysis activity (Figure 12) [223]. Lactose was converted to the trisaccharide GM3 using α2-3 sialyltransferase 1 (M144D) from *Pasteuralla multocida*, followed by a second α3,8-sialylation in 85% yield to GD3 fusing α2-3/8-sialyltransferase from *Campylobacter jejuni*. Subsequently, the quantitative enzymatic β1-4-GalNAc coupling to GD2 with β1-4-GalNAc transferase from *Campylobacter jejuni* followed by β1-3-Gal transfer with β1-3-galactosyltransferase resulted in GD1b with an overall isolated yield of 73% [223].

## 4. Reactor Engineering for (Non)-LeLoir Glycosyltransferases

One of the advancements in integrated biocatalytic processes using glycosyltransferases is the development of automated enzymatic synthesis, using either immobilized substrates or enzymes. Immobilized substrates allow for the spatiotemporal control of the produced glycoconjugate or oligosaccharide in a reactor. Two prominent approaches using immobilized substrates exist: (i) enzymatic *solution-phase synthesis* with tagged products allowing for rapid purification and (ii) enzymatic *solid-phase synthesis* on the surface of an insoluble carrier with soluble substrates and products.

**Figure 13:**
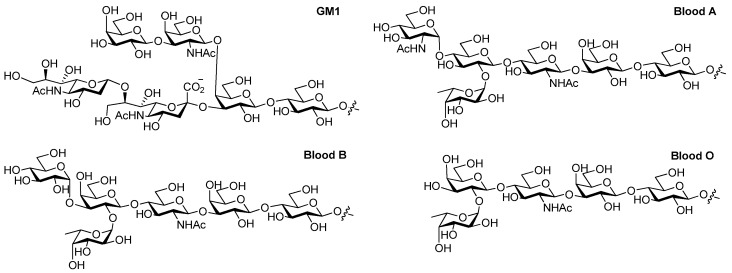
Examples of oligosaccharides synthesized with automated enzymatic synthesis where GM1 is a well-known ganglioside, and the antigens of blood types A, B, and O.

*Solution-phase synthesis* with a substrate bound to water-soluble [224] or thermo-responsive polymers [225], fluorous- [217,226,227,228,229,230,231,232,233,234,235,236], ion exchange [237], or lipid-like tags [238] has attracted much interest since it can bypass compatibility issues between enzymes and solid carriers. The main disadvantage of solution-phase assembly of oligosaccharides catalyzed by glycosyltransferases is the low catalytic efficiency and affinity for the substrates due to different steric and stereoelectronic properties induced by the substrate-bound tag. Automated enzymatic synthesis of oligosaccharides with Leloir glycosyltransferases has emerged as a promising approach with the thermoresponsive polymer poly(*N*-isopropylacrylamide) (PNIPAM) as a soluble or insoluble support of the sugars, allowing for the synthesis of the antigen of blood groups A, B, and O, as well as the production of the ganglioside GM1 in microchannel reactors (Figure 13) [239]. The Wong group reported a variety of water-soluble polymers of PNIPAM attached to carbohydrates with different linkers to minimize deleterious effects of the presence of the support on the activity of enzymes [24]. As a disadvantage, the covalent attachment of oligosaccharides to PNIPAM requires cleavage of the oligosaccharide with hydrogen peroxide (1M, pH 10), conditions which are incompatible with oxidative labile carbohydrates (i.e., thiosugars).

**Figure 14:**
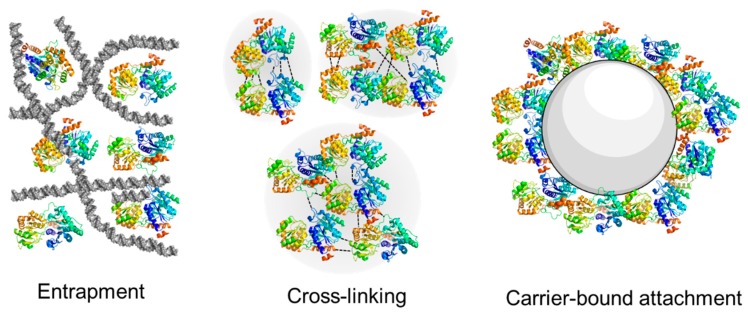
Different modes of immobilization of Leloir glycosyltransferases with entrapment, cross-linking, or carrier-bound attachment.

Alternatively, substrate bound *solid-phase synthesis* strategies also received attention [240,241]. The sugar is immobilized on the solid carrier, while the enzyme and sugar nucleotide donor are dissolved in the mobile phase [242,243,244,245]. Requirements for the full automatization of immobilized substrates are dependent on the development of (i) efficient enzymes; (ii) availability of glycosylation donors; (iii) the use of carrier and support material and (iv) linkers, spacers, and tags [24]. Continuous biocatalytic processes using *immobilized enzymes* are from an engineering perspective highly attractive due to the ease of reuse of the Leloir glycosyltransferase [189,246,247,248,249,250,251,252,253,254,255]. The immobilization of Leloir GTs enables a simplification of the reactor’s structure and allows for precise control of the enzymatic glycosylation process [255,256,257]. The immobilization of glycosyltransferases has been achieved by attachment onto *solid supports* [78,128,258,259,260], *entrapment* inside a porous carrier [252,261], or *cross-linking* in larger aggregates (CLEA) [262], as is shown in Figure 14. Furthermore, after immobilization the reusability, thermal, pH, and operational stability of the enzymes was often increased [128,262,263]. In particular cases, enzyme immobilization even created a more favorable micro-environment for enzyme activity [264] and selectivity [265,266].

On the other hand, the reaction conditions in a continuous biocatalytic process with immobilized enzymes can be harsh from an engineering point of view, due to vigorous mixing, high pressures, and flow rates. High enzyme stability of Leloir glycosyltransferases is required to tolerate shear stress [267,268]. Also, due to the lack of an universal enzyme immobilization technique many factors must be considered [256,268,269], including mode of *interactions* (i.e., enzyme-substrate/product, enzyme-carrier, substrate/product-carrier), *compatibility* of the carrier to reaction conditions (solvent, temperature, pH, etc.), and the *type of reactor or process* (i.e., batch reactor, packed-bed reactor, basket reactor, microfluidic reactor).

### Reactor Design for Glycosyltransferases

For industrial applications, besides the appropriate immobilization of the enzymes, the chemical character of the carrier’s surface is of high importance as are the particle size and pore structure which need to match the type of reactor: i.e. tank vs. tube/column and the mode of operation: periodic/batch vs. continuous flow. Conventional stirred tank reactors (STR) [259,266,270,271] are still frequently applied, but basket and in particular rotating bed reactors [272] are increasingly used in batch operations. In continuous flow applications, packed-bed (micro)reactors [273] have been most popular, but structural (micro)reactors [274,275], lab-on-a-chip [276], and capillary microreactors are on the rise [277]. The STRs are predominantly used in biotechnology owing to cost efficiency and versatility. However, vigorous mixing results in frequent collisions of fine biocatalyst particles resulting in tensile and shearing forces which enhance abrasion or disintegration of the enzyme or its carrier [272]. Moreover, the mixing on a medium and large scale can be insufficient to prevent “hot spot” formation, resulting in enzyme denaturation. But even more importantly, as mass transfer of reactants to fine particles of the biocatalysts or freely suspended enzymes is low, the mass transfer does not keep up the pace of intrinsic activity of the highly active enzymes expressed by turnover frequency values of about or over 10^4^ s^−1^. In effect, it is the mass transfer that strongly impacts, or even fully controls the apparent rate of the enzyme catalyzed reactions carried out in STRs, and that has unfortunately often been overlooked [277,278]. The recovery of the freely suspended biocatalyst particles can be challenging, requiring filtration or centrifugation [279]. The biocatalyst recovery can be simplified using a basket-rotating bed reactor (RBR), which enables simultaneous mixing and effective percolation of liquid through the bed of the catalyst packed in a cylindrical basket, thus avoiding the catalysts destruction, enhancing mass transport, and facilitating separation of the catalyst [280,281,282]. However, the size of the applied catalyst particles/enzyme carriers needs to be larger than 0.1–0.2 mm [280,281]. The scale-up of RBRs is challenging due to the large size of the rotor as well as the power required for rotating, although a 750-L scale has been successfully demonstrated (Chiralvision. Low-volume continuous flow reactors with flow through channels typically in the range of diameters 0.1–0.5 mm (capillary microreactors) are better scalable [277,283,284,285]. The problems related to scaling up are resolved by numbering up the (micro) reactors in a parallel process, designated as ‘scaling out’. In particular, if thermal effects are not too strong, as is often the case, for the same type of packing the scalability is not a problem. Average linear velocity of flowing reactants and a mean residence time need to be identical on different scales to obtain the same conversion. In addition, owing to narrow channels the time needed for substrate transfer from the center of the liquid channel to the wall-attached catalysts is significantly reduced and the transfer of heat and mass increased, with a positive effect on the apparent reaction rate. Also, the ratio of the active surface of the reactor to its volume may be increased by a factor of 50 or even much more, (e.g., from about 10^3^ m^−1^ in industrial reactor to ~5 × 10^4^ m^−1^ for 0.1 mm capillaries) which results in an increase of volumetric productivity (space-time-yield) and hence more profit and lower investment costs [277,278,285]. Moreover, the low reaction volume favors the reduction of potential hazards, particularly important in the case of highly exothermic reactions or when hazardous substances are involved [278,284]. A simplified approach may be used to evaluate process boundaries for the capillary microreactors with enzymes attached to the wall surface [277]. But more in-depth analyses of design and modelling issues for various continuous flow microreactors have also been reported [285].

**Figure 15:**
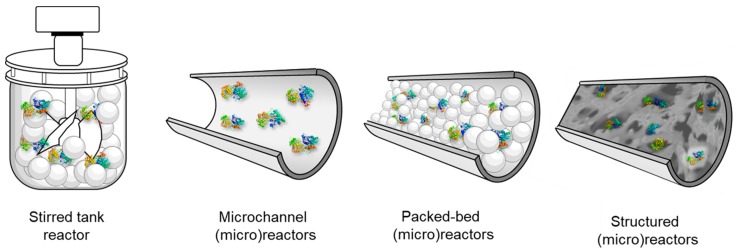
Different reactor types for the immobilization of Leloir glycosyltransferases.

Different examples of reactors that have been used with glycosyltransferases are shown in Figure 15. *Stirred tank reactors* are flexible in design and operation conditions, but often require high operation costs and vary in the product quality per batch [128,259,266,270,271]. *Microchannel reactors* feature flow-through channels of micrometric sizes that contain the enzyme immobilized on their wall surface [277]. *Packed-bed (micro) reactors* contain fine particles with immobilized enzymes in a flow-through channel, allowing for a higher volumetric activity than microchannel reactors. The heterogenous biocatalyst should not be able to compact to avoid high pressure drops, while mass transport between liquid reactants and catalyst surface is enhanced owing to a more chaotic flow which facilitates mixing. The large pressure drop, even at low flow rates may, however, be a problem if fine catalyst particles are applied. Structured microreactors contain a reactor core made of a porous monolithic structure with open, usually curved pores/channels connected with each other offering excellent mixing and mechanical stability. Moreover, the pressure drop can be significantly reduced and flow rate increased, compared to the packed-bed reactors, and this boosts productivity. The enzymes are immobilized either on the external surface of the monolith or in its pores [273].

A packed-bed reactor is a commonly used system for continuous production with a heterogeneous biocatalyst, especially because one can immobilize simultaneously different enzymes. Schöffer et al. used glutaraldehyde-activated chitosan spheres [259] and amino- or thiol- functionalized silica particles [260] as a support for cyclodextrin glucosyltransferase immobilization. The silica-based biocatalyst was successfully applied in a packed-bed reactor for continuous cyclodextrin production and maintained 100% of its initial activity after 200 h, whereas activity of the chitosan-based catalyst decreased to 78% of its initial value already after 50 h. This was ascribed to the super packing of spheres, resulting in the reduction of bed height by 45%, and thus in a decrease in the residence/reaction time. However, after washing and re-packing, the spheres recovered 100% of their initial activity.

An interesting effect was observed by Cho, et al. [271], who compared the performance of batch and continuous packed-bed reactors using Eupergit C250L as an enzyme support. The batch reaction was performed for trehalose production from maltose using trehalose synthase for more than 20 h. They found that the product composition was almost the same after 10 h and a maximum trehalose production yield of 25% was established. Trehalose production was improved using a packed-bed bioreactor, wherein the yield reached 42% with a retention time of 100 min. The authors claimed that continuous feeding of fresh substrate into the packed-bed reactor might have eliminated and removed inhibitory compounds from the solution such as by-products formed and accumulated during the batch reaction and thus increased the production yield. A combination of continuous-flow stirred tank reactor (CSTR) with a packed-bed reactor (PBR) was also studied [270]. A highly concentrated starch solution was first partially converted to β-cyclodextrins in a CSTR, which resulted in a decrease of starch viscosity. After that, the reaction mixture was pumped through the PBR. The integrated reactor offered much higher concentration of the final product than each of the reactors separately.

Integration of two or more reactors attracts increasing attention, especially if two or more enzymes are applied. For continuous flow nucleoside synthesis Cattaneo, et al. [273] combined a PBR, filled with purine nucleoside phosphorylase immobilized on silica particles, with uridine phosphorylase immobilized on a silica monolith. In the first approach, co-immobilization of both enzymes on a slightly longer silica-filled PBR was tested, and a high immobilization yield was obtained. However, a very high backpressure of the system (>10 MPa) was registered even at a low flow rate value of 0.1 mL/min, thus hampering the full characterization of the reactor system and resulting in a dramatic drop in conversion. Nonetheless, the application of a monolithic reactor, which exhibited only 6 MPa of pressure drop at a flow rate of 0.5 mL/min, combined with a shorter PBR, showed good activity and stability [273]. The additional advantage of this set-up would be the availability of a single bioreactor that could be used independently, either for “one-enzyme” synthesis or in a different sequence.

**Figure 16:**
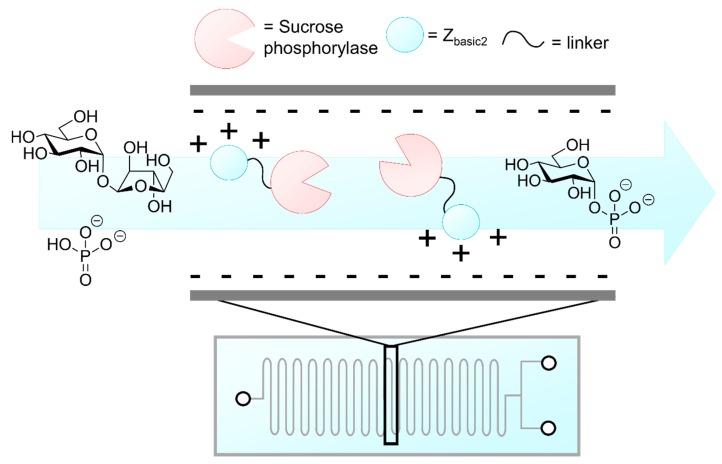
Glass microchannel reactor with immobilized enzyme sucrose phosphorylase attached to the siliceous wall [277]. Attachment occurs via the highly positively charged Z_basic2_ binding module, which binds to the negatively charged silica surface.

Recently Nidetzky, et al. [277], presented an elegant exemplary glycosylation process with sucrose phosphorylase immobilized on the internal surface of a microchannel (Figure 16). Its mathematical model clearly demonstrated that microreactors with the lower hydrolytic channel diameter (*d*_h_) exhibit enhanced performance in terms of conversion and space-time-yield (STY). As the enzyme was attached on the microchannel’s wall only the external mass transfer had to be considered [285], and the enzymatic transformations appeared to experience a shift from diffusion to reaction control with miniaturization of *d*_h_ (second Damköhler number—Da_II_ < 1). Thus, the microreactors, in consequence of their small *d*_h_, emerge as an effective means of gaining full control of the reaction [277]. However, the practical limits to the decrease in *d*_h_, due to high pressure drop, and increased tendency of microchannel clogging have to be kept in mind [274,277]. Therefore, to boost both STY and microreactor performance, a combination of *d*_h_ decrease and enzyme activity increase appears to be the rational solution [277].

To summarize, the application of miniaturized synthetic systems and flow microreactors with immobilized enzymes in particular, attracts attention for the synthesis of more complex carbohydrates. The reported studies clearly demonstrate important advantages of microreactor-based synthetic processes: good stability and high activity that allow for very effective/highly productive syntheses of targeted carbohydrates. While the application of capillary or packed-bed microreactors has been best characterized, structured microreactors are emerging as a class of miniaturized devices that offer additional advantages.

## 5. Summary and Outlook

The discovery, characterization, and engineering of Leloir glycosyltransferases has expanded the synthetic toolbox to couple, elongate, or branch glycoconjugates, oligosaccharides, and glycans with high regio- and stereoselectivity. Efficient regeneration systems and large-scale production of nucleotide sugar donors under either kinetic or thermodynamic control increased the efficiency of enzymatic glycosylation, reducing overall process costs and the use of stoichiometric amounts of nucleotide phosphates. In this review, the importance of the thermodynamics of glycosylation reactions has been given attention, separating kinetics from thermodynamics for the coupling of a wide variety of NDP sugar donors with acceptors, including sacrificial phosphate donors for NDP (re)generation.

The wide range of different Leloir glycosyltransferases has allowed for the implementation of enzymes in glycochemistry, and consequently, industrially applicable glycosylation methodologies are now in progress. The protein production improved significantly by using (fluorescent) solubility tags, allowing for the production of the biocatalyst in high(er) titers. Despite of the production of enzymes becoming a routine, no industrial application of Leloir glycosyltransferases for the glycosylation of large oligosaccharides has yet been scaled to large volumes, in contrast to non-Leloir glycosyltransferase (i.e., cyclodextrin glycosyltransferases). Until now, one of the main limitations has been the cost-efficient production of NDP sugar donors, which has been an important topic of interest in the last decade. Indeed, due to the advance of many chemical and biocatalytic NDP-sugar production processes, their commercial cost price has been rapidly declining over the last few years.

As the demand for high-value antigens is increasing, the number of biocatalytic glycosylation processes applied for the synthesis of these complex oligosaccharides can be anticipated to rise. One trend is the embracement of automated enzymatic synthesizers for the computer-controlled synthesis of large oligosaccharides using Leloir GTs. In comparison to non-enzymatic coupling strategies, which mostly rely on protection group chemistry, Leloir GTs have now been shown to couple a wide spectrum of unprotected sugar acceptors with excellent regio- and enantioselectivity. Due to the availability of the nucleotide sugar donors and well-established NDP (re)generation systems, Leloir GTs matured as a competitive glycosylation strategy for the enzymatic synthesis of oligosaccharides.

Future developments for industrial enzymatic glycosylation are expected to mostly be focused on optimizing the overall glycosylation process conditions. The main drivers for selecting the most optimal process can be attributed to economic (i.e., revenue of products), development (i.e., time), and process (i.e., performance) parameters. Multiple aspects influence these important aspects, such as the selection of the most optimal reactor design, separating batch versus continuous process operation, the choice of either immobilized substrates to immobilized enzymes, or the use of NDP-regeneration system or stoichiometric use of NDPs. Different enzymatic solution-phase and solid-phase glycosylation strategies have been developed for the automated enzymatic synthesis of carbohydrates. The enzymatic synthesis of an immobilized substrate allows for the purification to become more straight-forward, but requires the stoichiometric use of enzymes. On the other hand, immobilized Leloir GTs in continuous operations have been described sparingly in cascade glycosylation reactions. Immobilized enzymes, ensuring process flexibility and the purification of the produced oligosaccharide are engineering design challenges which have yet to be met.
